# Pneumoconiosis computer aided diagnosis system based on X-rays and deep learning

**DOI:** 10.1186/s12880-021-00723-z

**Published:** 2021-12-08

**Authors:** Fan Yang, Zhi-Ri Tang, Jing Chen, Min Tang, Shengchun Wang, Wanyin Qi, Chong Yao, Yuanyuan Yu, Yinan Guo, Zekuan Yu

**Affiliations:** 1 Department of Radiology, The Affiliated Hospital of Southwest Medical University, Taiping Street, Luzhou, 646000 Sichuan China; 2 School of Physics and Technology, Wuhan University, Wuhan, 430072 China; 3 Key Laboratory of Industrial Dust Prevention and Control and Occupational Health and Safety, Ministry of Education, Huainan, China; 4 Department of Radiology, Huashan Hospital, Fudan University, No.12 Wulumuqi Road (Middle), Shanghai, 200040 China; 5 Luzhou Center for Disease Control and Prevention, Luzhou, 646000 Sichuan China; 6 School of Information and Control Engineering, China University of Mining and Technology, Xuzhou, 221116 China; 7 Academy for Engineering and Technology, Fudan University, Shanghai, 200433 China; 8 Guangxi Key Laboratory of Automatic Detecting Technology and Instruments (Guilin University of Electronic Technology), Guilin, China

**Keywords:** Pneumoconiosis diagnosis, X-rays, Deep learning, U-Net, ResNet

## Abstract

**Purpose:**

The objective of this study is to construct a computer aided diagnosis system for normal people and pneumoconiosis using X-raysand deep learning algorithms.

**Materials and methods:**

1760 anonymous digital X-ray images of real patients between January 2017 and June 2020 were collected for this experiment. In order to concentrate the feature extraction ability of the model more on the lung region and restrain the influence of external background factors, a two-stage pipeline from coarse to fine was established. First, the U-Net model was used to extract the lung regions on each sides of the collection images. Second, the ResNet-34 model with transfer learning strategy was implemented to learn the image features extracted in the lung region to achieve accurate classification of pneumoconiosis patients and normal people.

**Results:**

Among the 1760 cases collected, the accuracy and the area under curve of the classification model were 92.46% and 89% respectively.

**Conclusion:**

The successful application of deep learning in the diagnosis of pneumoconiosis further demonstrates the potential of medical artificial intelligence and proves the effectiveness of our proposed algorithm. However, when we further classified pneumoconiosis patients and normal subjects into four categories, we found that the overall accuracy decreased to 70.1%. We will use the CT modality in future studies to provide more details of lung regions.

## Introduction

Pneumoconiosis is an occupational lung disease where a typical pathological change is diffuse interstitial lung fibrosis. It is caused by the long-term exposure of workers to productive mining dust during their occupational activities [1]. According to statistics from the Chinese health department, as of 2018, more than 975,000 cases of occupational diseases have been reported nationwide, with occupational pneumoconiosis accounting for nearly 90% and more than 870,000 cases. In addition, pneumoconiosis caused by hazardous dust is a progressive disease, which can still occur after exposure ceases [2, 3]. As a result, it is foreseeable that many new cases of pneumoconiosis will continue to occur for a long time to come. However, currently there is no cure for pneumoconiosis. Hence, minimizing exposure to harmful dust particles and detecting the disease at an early stage are by far the most effective interventions [2].

The core of X-ray based pneumoconiosis diagnosis is the accurate interpretation of chest radiographs [4]. However, this is a relatively challenging task because the image features indicating pneumoconiosis on the digital chest radiographs are often difficult to capture and identify. In other words, there is a lack of pneumoconiosis diagnostic doctors relative to the heavy workload of pneumoconiosis detection. Recently, artificial intelligence (AI) has achieved great success in medical imaging tasks [5]. As a subset of AI, machine learning has been applied in radiomics analysis to build radiomics signatures and predictive models [6, 7]. In this thread of emerging literature, machine learning is used to evaluate early esophageal adenocarcinoma [8], classify focal liver lesion [9], predict the degree of histological tissue invasion and patient survival in lung cancer [10], classify lung nodules [8], diagnose and classify COVID-19 [11–15]. A number of scholars have also conducted a lot of research on the application of AI in the diagnosis of pneumoconiosis, including classification [16, 17], detection [18] and so on. However, most of them used a very limited number of cases. For instance, Okumura et al. [17] proposed to classify the pneumoconiosis as category 0 to category 3, but only a total of 55 images were used in their analyses. On the other hand, most studies [19, 20] considered data with an uneven distribution of pneumoconiosis cases across stages, so the data can only be divided into two categories, namely normal and pneumoconiosis, in which case, the model can only be used to determined whether if the patient has pneumoconiosis.

To overcome the limitations of the existing methods, in this paper, we extract the lung fields on both sides of the pneumoconiosis digital radiography (DR) chest radiographs using the U-Net model based on deep learning. We use ResNet to learn the image features to obtain an accurate classification of the pneumoconiosis grades and explore the application of artificial intelligence in pneumoconiosis detection. Our main contributions are as follows: We collect and create a new dataset of the largest number of pneumoconiosis data currently available. We present a new deep learning pipeline for the pneumoconiosis quadruple classification task, where focal loss is used for the first time for pneumoconiosis detection. In addition, we also use the model parameters trained in the public dataset for the region of interest extraction in this paper to help further improve the performance of our model for the classification task.

In summary, we have proposed a new medical model for pneumoconiosis detection and validate it on the real data collected in practice. We present results that show the effectiveness of our proposed computer aided diagnosis system.

## Methods and materials

### Patient characteristics

In our study, all the procedures abided by the protocol of Affiliated Hospital of Southwest Medical University. Ethical approval was obtained from the Ethical Committee of the hospital (KY2020200). The institutional review board approved this retrospective study and waived the need to obtain informed consent from patients. A database of 1760 anonymized digital radiography (DR) posteroanterior chest radiographs (CXR) showing evidence of pneumoconiosis was generated from January 2017 to June 2020. Age 20–95, 259 cases are female, and the rest are male. Their duration of exposure to dust ranged from 2 to 29 years, with an average of 6.2 ± 5.6 years. There are 1248 positive cases and 512 negative cases. We present patient demographics information in Table 1.

**Table 1:** Data summarization

	Pneumoconiosis (n = 1248)	Normal (n = 512)
N, Male (%)	1237 (99.1)	264 (51.6)
Age, mean years (SD)	59.0 (9.3)	37.5 (11.6)
Time of dust exposure (y)	2–29	2–6
Category		
0	–	512
1	352	–
2	478	–
3	418	–
Subgroups		
Per training	1123	461
Validation	114	51

The inclusion criteria included: (1) All patients with a history of exposure to dust; (2) Patients with chest radiographs, which are of good, or at least acceptable quality according to the guidelines of the ILO; (3) All positive cases have Certificate of diagnosis by the unit with pneumoconiosis diagnosis qualification. The exclusion criteria included: Subjects with lung or pleural disease, which affect the diagnosis and the grading of pneumoconiosis, such as with those with pneumothoraxes, pleural effusions, or partial or total resection of lung tissue on one side.

### Chest radiographs acquisition

Chest radiographs were obtained using different DR scanners, including SHIMADZU 0.6/1.2P324DK-85/UNITED IMAGING uDR 770i /WDM 1000MD, tube voltage of 100-125kVp, exposure time: < 100 ms; source image distance (DIC): 180 cm; Photography position: standard chest posteroanterior position, upright position. These images were calibrated to meet the Digital Imaging and Communications in Medicine (DICOM) standard.

### Chest radiographs analysis

Following the guidelines of the ILO, an expert panel formed by a certified radiologist and four occupational physicians reviewed these images. They recorded features include small opacities, large opacities, and Pleural abnormalities. Small opacities are described by profusion, affected zones of the lung, shape (rounded or irregular) and size. In addition, asbestos exposure history, pleural abnormalities, including pleural plaques (localized pleural thickening), costophrenic angle obliteration and diffuse pleural thickening, are regarded as the most important evidence for classifying pneumoconiosis in diagnosis. Finally, all patients are classified into four categories, namely 0 (n = 512), 1 (n = 352), 2 (n = 478) and 3 (n = 418).

### Method overview

**Fig. 1:**
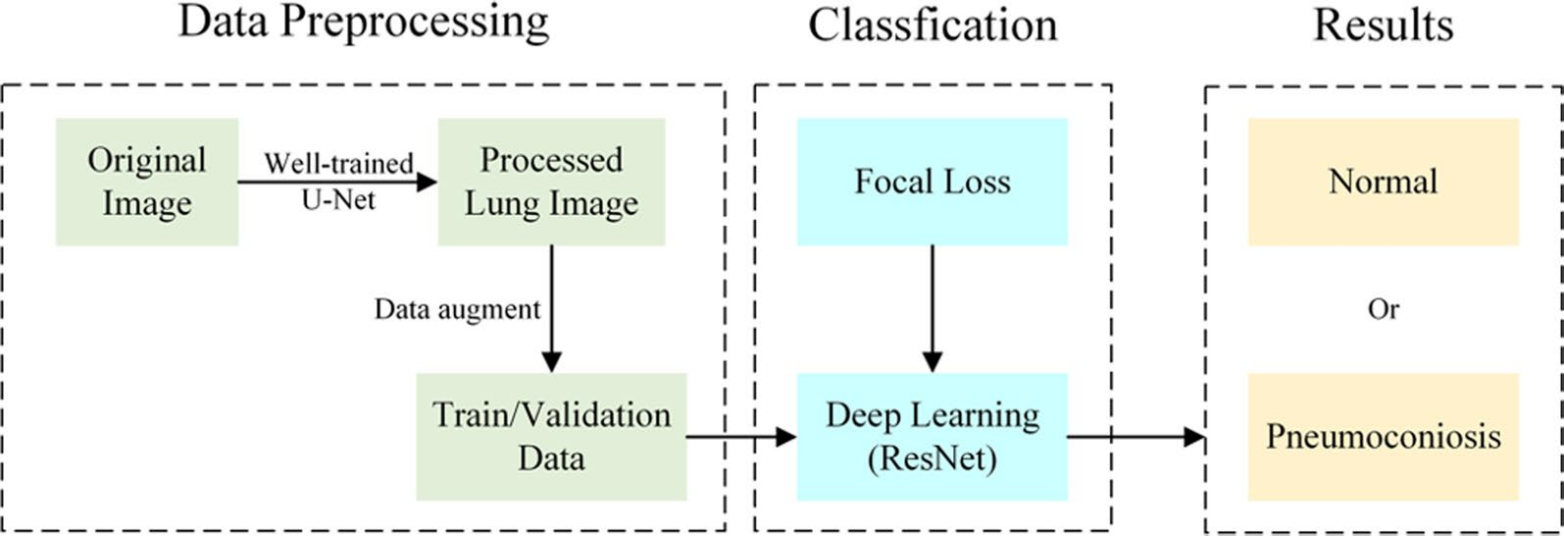
An overview of the method adopted in this work

The overview of the method used in this paper is shown in Fig. 1, which includes three main parts. The first part of the method is data pre-processing, which consists of segmenting our own chest X-ray images using parameters trained by U-Net [21] in a publicly relevant dataset to obtain segmentation labels and processing the dataset using ten different data enhancement methods to perform 10-fold crossover experiments. The second part is to use the state-of-the-art deep learning framework ResNet [22] and replace the original loss function with focal loss for classification. The third part shows the classification results, which we have improved to a quadruple classification compared to the previous ordinary binary classification model.

### Data preprocessing

U-Net [21], which is proposed for medical image segmentation, has achieved great success in many areas and has been shown to be effective for different types of medical images. The two main features of U-Net are the U-shape structure and the skip connection. U-Net's encoder down samples four times consecutively for feature extraction and size halving. Symmetrically, its decoder up samples four times accordingly to restore the high-level semantic feature map obtained by the encoder to the resolution of the original image. During the up-sampling process, the symmetric structure feature maps were stitched together by skipping connections to reduce the semantic divide.

In this work, the labels of our chest X-ray images only include diagnosis results. Accurate segmentations of the images would typically require intensive manual labor of professional doctors. To obtain the segmentation of the lung field in our datasets, we train a U-Net using a dataset of chest X-ray images on Kaggle dataset, with standard labels and lots of training data.

**Fig. 2:**
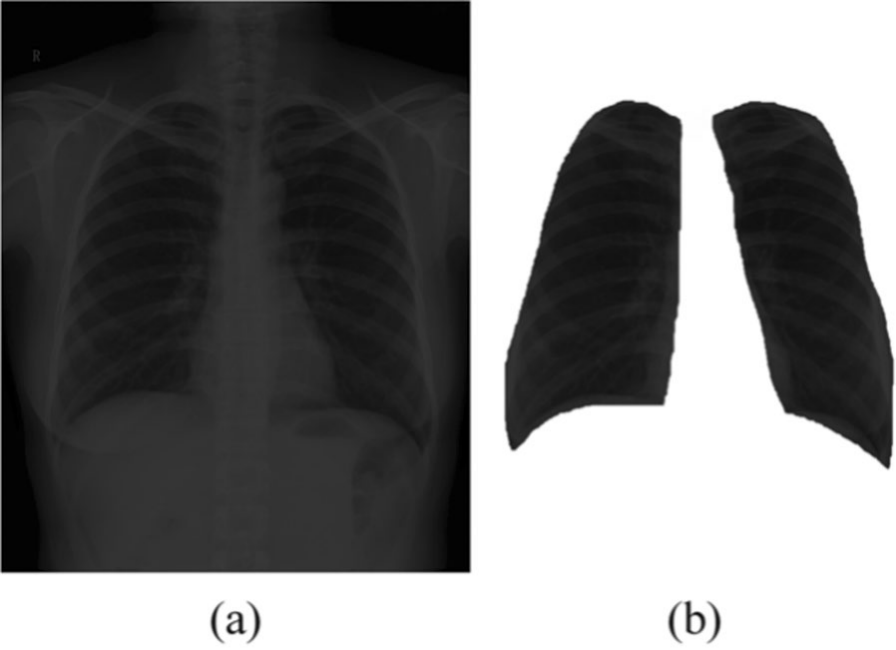
An example of **a** original image and **b** that after lung region segmentation

An example of the segmentation is shown in Fig. 2, where (a) is an original image and (b) is the segmentedlung field. After lung field segmentation, a data augment process is presented to obtain more images for training. First, 10-fold cross-validation is adopted for dividing the original dataset into training and validation dataset. There are a total of 1760 patients, among which, 176 patients are used for validation. In addition, to augment the training dataset (1584 patients each time), we vary the brightness of each training image and present eight levels of different brightness of training images. This results in a total of 12,672 training images obtained each time.

### Classification

As a powerful technology in image processing and computer vision, deep learning has been applied in many different areas, such as e-diagnosis of brain intracranial hemorrhage using CT images [23], detection and classification of multi-class skin lesion [24, 25], prediction of COVID-19-pneumonia [26], and Classification of positive COVID-19 CT scans [27]. In this work, ResNet-34 [22],which has 34 layers in total, is adopted for classification tasks. ResNet uses residual learning methods to extract more information from its deep structure. Some layers with residual function rather than no-referenced functions are presented, using a shortcut connection to add the last one or several layers with this layer. The shortcut connection in the residual module allows ResNet, which is one of the landmarks in the deep learning field, to achieve an improved classification accuracy with considerably increased depth. Besides, the number of training images in two different classes, normal and pneumoconiosis, has a considerable gap, which is also described as the class imbalance problem in the artificial intelligence field and always leads to bad performance. In traditional binary classification tasks, the cross-entropy loss is commonly applied, which will give the same weights for each training sample. However, due to the class imbalance problems in two categories, the cross-entropy loss will ignore a small number of difficult samples. To solve this issue, a focal loss [28] is adopted as the loss function of the deep learning framework, which can promote the weights of categories with smaller samples effectively. In addition, the focal loss can help to de-weight the well-classified samples in the loss function of the entire learning systems. Through some parameters in focal loss, a balance between two categories can be obtained, which can efficiently improve the binary classification accuracy. Its mathematical form is shown below:

1 $$\begin{array}{*{20}c} {Loss_{Focal} = \left\{ {\begin{array}{*{20}l} { - \partial \left( {1 - y^{\prime}} \right)^{\gamma } logy^{\prime}, } \hfill & {y = 1} \hfill \\ { - \left( {1 - \partial } \right)y^{^{\prime}\gamma } log(1 - y^{^{\prime}} ),} \hfill & {y = 0} \hfill \\ \end{array} } \right.} \\ \end{array}$$

Furthermore, the 10-fold cross validation is adopted in this study, where there are a total of 1760 patients and 176 patients are used for validation in each round. After data augmentation mentioned in the above section, a total of 12,672 training images are obtained during each round. For the implementation details, both training and validation processes are carried out by Pytorch in one RTX 2080Ti GPU. Training batch size is 32, the learning rate is 0.0001, the number of training epoch is set to 50, and the output number of classes is 2.

### Evaluation

To better evaluate the classification performance on pneumoconiosis, several evaluation metrics are used in this work. Classification accuracy on training and validation sets with training epochs is adopted, while the loss with training epochs is also given to show the training details of deep learning. Receiver operating characteristic curve (ROC) is a tool used to measure unevenness in classification. Area under curve (AUC) is defined as the area under the ROC Curve. They are often used to evaluate the merits of a classifier. The abscissa of ROC curve is false positive rate (FPR), and the ordinate is true positive rate (TPR). Their mathematical formulas and meanings are shown as follows:

2 $$\begin{array}{*{20}c} {TPR = \frac{TP}{{TP + FN}}} \\ \end{array}$$

3 $$\begin{array}{*{20}c} {TPR = \frac{{{\text{FP}}}}{{{\text{FP }} + {\text{ TN}}}}} \\ \end{array}$$

## Results

**Fig. 3:**
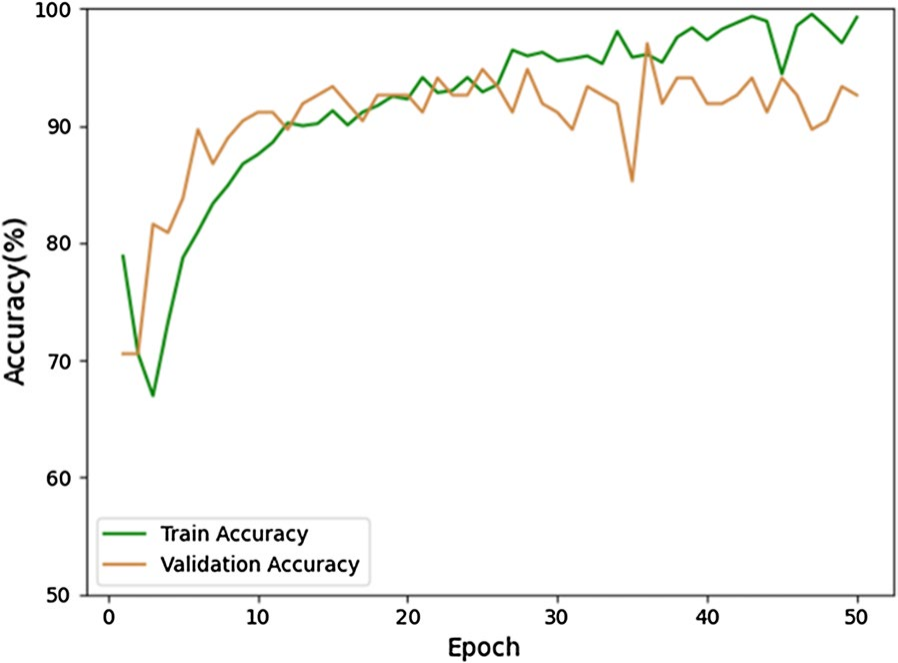
The training and validation accuracy with epochs

**Fig. 4:**
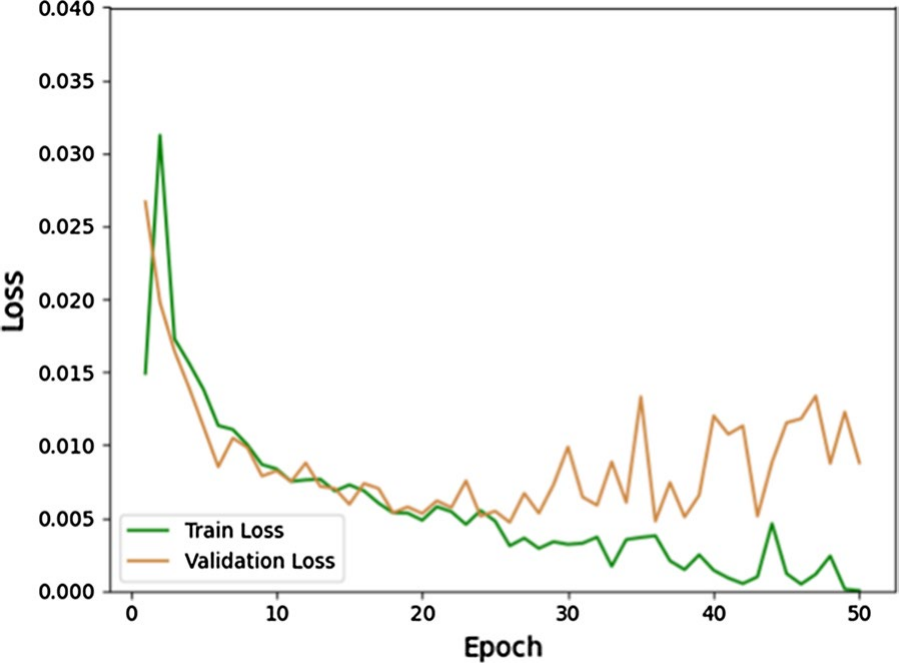
The training and validation losses with epochs

**Fig. 5:**
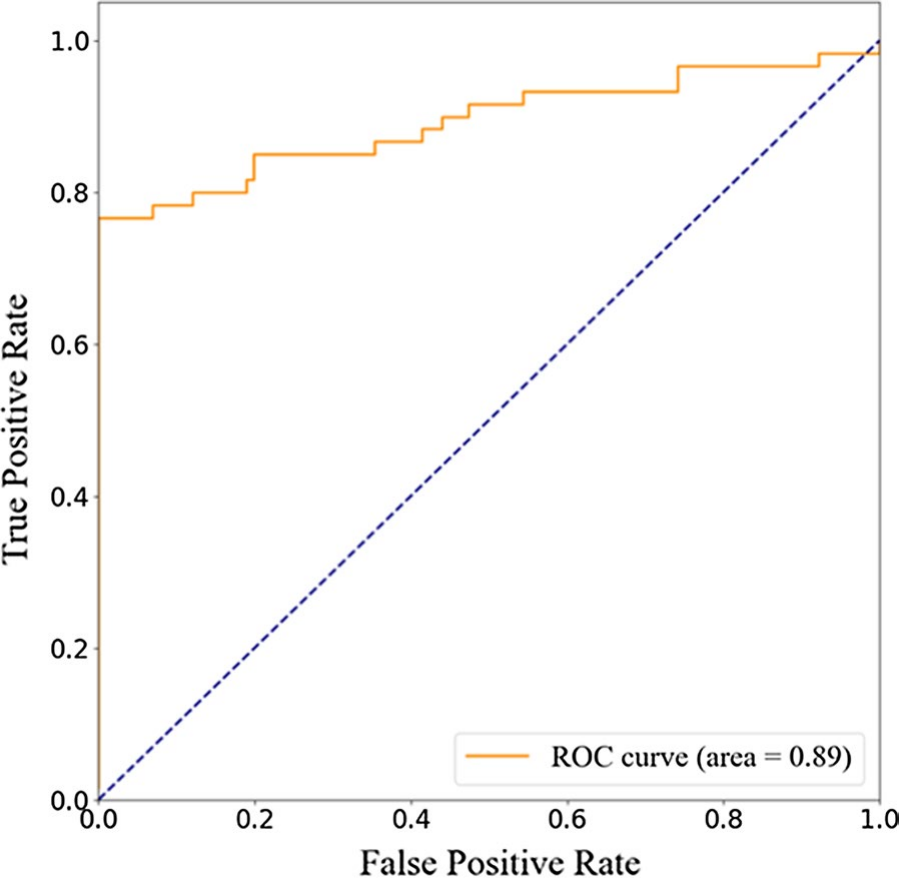
The ROC curve

The training and validation accuracy with epochs are shown in Fig. 3, and the loss curves are shown in Fig. 4. It can be seen from Fig. 4 that the validation loss curve reaches its lowest point after epoch 20. Hence, the validation accuracy curve is also stable after epoch 20. If we take the average accuracy between epoch 21 and epoch 50, we can get a validation accuracy of 92.46%. Specifically, the highest accuracy obtained is 96.14% and the lowest is 84.66%, where the confidence interval of accuracy is 92.46 ± 7.8%. The ROC curve of validation and its AUC is shown in Fig. 5, which shows the AUC is 0.89.

## Discussion

Machine learning plays an important role in the detection of lesions, especially when the number of patients is rising and the doctors are unable to provide adequate support in which case, it is particularly important to build a reliable identification and detection model. Although the use of deep learning algorithms for pneumoconiosis diagnosis is not new, the number of cases considered in most studies has been limited. For instance, X Wang et al. [19] used a total of 1881 DR images in their analyses. They compared the performance of two experienced radiologists in interpreting pneumoconiosis, who respectively had more than five- and ten-years’ experience. They had an AUC of 0.668 and 0.772, respectively. The more experienced radiologist had a relatively better performance. Their studies involved DR examinations collected from a single institution using the same DR imaging machine. In this study, the dataset of 1760 DR chest radiographs comes from multiple centers and multiple devices, and the research results are more comprehensive. Besides, to our knowledge, our dataset is larger than those considered in the past investigations [16, 17, 20, 29].

In addition, a comparison with state-of-the-art works is given in Table 2, where 11 different algorithms are adopted for pneumoconiosis diagnosis [30]. The binary classification results of our proposed method significantly outperform those found in the aforementioned works, where the second-best result in these works, obtained from a general neural network classifier, is only 83%.

**Table 2:** Comparison of accuracy in pneumoconiosis diagnosis with different algorithms

Machine learning classifiers	Accuracy (%)
OC-SVM-RBF with raw CXR	73.17
Isolation Forest with raw CXR	68.29
Autoencoder and feed-forward NN with raw CXR	68.29
MLP	71.77
SVM-RBF with raw CXR	68.29
Autoencoder and SVM-RBF with raw CXR	73.17
NN	83.00
Linear- SVM	74.80
KNN	69.30
Ridge Classifier	76.90
Random Forest	70.80
Res-Net (Our method)	92.46

**Fig. 6:**
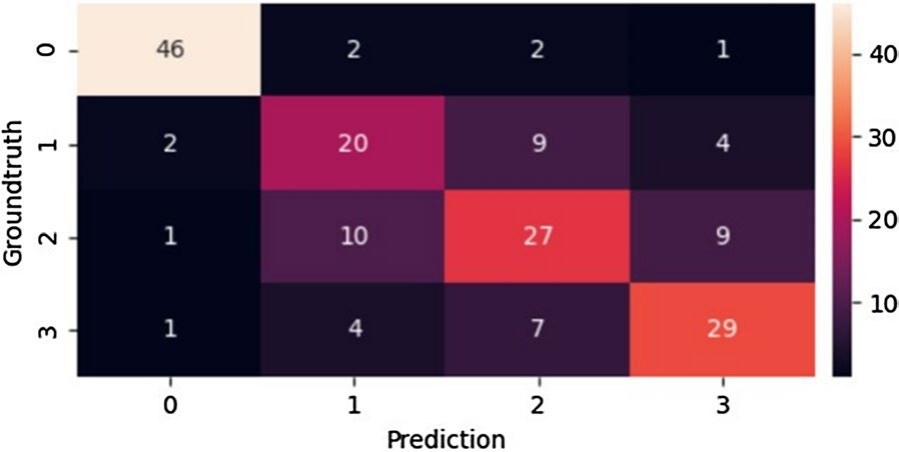
Confusion matrix of pneumoconiosis classification into four categories

There are also some limitations in this study. First, the dataset in this study is relatively small for deep learning. However, as shown by our statistical analysis on the independent test set (*p* < 0.01), this dataset is sufficient to verify the feasibility of deep learning in pneumoconiosis assessment. Second, the current dataset only contains chest radiographs. Although chest radiographs are the standard method for the diagnosis of pneumoconiosis, they cannot provide more details of the opacities regions in the lungs. This limitation can be overcome by the images of Computed Tomography (CT). Therefore, CT of pneumoconiosis should be considered in future studies, especially for the pneumoconiosis grading. Third, the experiments on the four-category classification of pneumoconiosis s are also conducted in Fig. 6 where a confusion matrix is given. It can be seen from Fig. 6 that the classification performance on categories 1, 2 and 3 is not ideal, with the entire accuracy being only 70.1%. We can observe that the X-ray modality does not provide sufficient details of features that reveal different grades of pneumoconiosis. To resolve this issue, CT modality will be considered in our future research.

## Conclusion

In this study, the automatic pneumoconiosis screening system has been developed. We propose a new preprocessing pipeline with the ResNet classification model, and show that the model achieves satisfactory performance. Compared to many existing studies, a larger test dataset is used in our study. While this pipeline is capable of reducing the workload of radiologists, we are aware of its current limitations in the actual application of pneumoconiosis diagnosis. We aim to explore models that can yield more accurate grading of pneumoconiosis in our future work.

## Data Availability

The datasets generated and analyzed during the current study are not publicly available dueto patient privacy and ethical requirements but are available from the corresponding author on reasonable request.
